# Book Reviews

**Published:** 1832-01

**Authors:** 


### CRITICAL ANALYSES.

Quæ laudanda forent, et quæ culpanda, vicissim

Illa, prius, cretâ; mox hæc, carbone, *notamus*.—PERSIUS.

*An Essay on the Epidemic Cholera of India.* By REGINALD ORTON, Surgeon H.P., late of his Majesty's Twenty-fourth Regiment of Foot. *Second Edition, with a Supplement.*—8vo. pp. 488. Burgess and Hill, London.

THE deep interest that has long been felt, both by the profession and the public, concerning every point that relates to cholera, has, within these few weeks, increased rather than diminished, in consequence of this direful malady having at last approached our own shores, and made its appearance at Sunderland, and other adjoining places. Independent, however, of the anxiety which medical practitioners must necessarily feel, both for the public welfare and their own interest, to become thoroughly acquainted with the nature and treatment of a disease with which it is at least probable they will have to contend, there are other circumstances which alone render these inquiries of much moment, and their elucidation of the utmost importance to the medical world: for, from the statements that have been given to us by the best authorities, it appears indisputable that cholera bears a very near relationship to many other serious maladies that often fall under our observation; and there can be no doubt that, while we instruct ourselves respecting its pathology and therapeutic means, we shall at the same time be enlarging our general views of those other morbid conditions and diseases to which it appears to be more or less closely allied.

The work before us, unlike some of those which have recently been "shot unfinished from the press," comes from one who has had ample opportunities of seeing the disease of which he treats. Cholera has formed the object of Mr. ORTON's earnest and deliberate inquiries, and the information he long ago contributed respecting it has been taken as a guide by some of our best systematic writers. We are quite aware that the attention of our readers may be exhausted by the repeated discussion of even the most interesting theme, and we will endeavour to carry them with us through the notice we shall take of Mr. Orton's work with as little unnecessary fatigue as possible, by very briefly touching upon those points which have already been dwelt upon in the accounts we have given of other treatises upon the same subject.

*Description of Cholera.* In conformity with other writers, we find from Mr. Orton, that the attack of this epidemic is sometimes sudden and violent, but, in a great majority of instances, not without some premonitory symptoms; as simple diarrhœa continuing for several days; extraordinary depression of spirits and general uneasiness, with tremor and sense of debility; giddiness, headach, sometimes pains in the limbs are felt; griping of the bowels and nausea. The circulation and temperature of the body are variously disturbed; but the pulse is commonly accelerated and weakened, and the skin is moist, and feels colder than usual. In general the severer affections quickly follow these precursory signs: the stools become frequent and watery, and of a peculiar smell, and greyish-white colour, like barley water; vomiting, followed by copious sweats, comes on; the anxiety and debility rapidly increase. The countenance assumes a very peculiar appearance, by which alone the disease may generally be distinguished: it bears a striking resemblance to the appearance of *age*, and seems to arise from the paleness, wasting, and shrinking of the features, and the depressed and disturbed state of the mind, giving an expression of care, anxiety, and alarm. It is usually during a fit of vomiting that spasms of the muscles are first felt; they are almost invariably of the tonic kind, and recur in short paroxysms, and are attended with excruciating pain. Mr. Orton has in no instance had occasion to believe that the spasms were injurious by interrupting respiration or circulation: he has often found the pulse sink at once on the first appearance of spasm, from the natural state, so as scarcely to be felt, or even disappear entirely from the wrist in an instant, and, when spasms do not occur, this fatal failure of the circulation often appears with the first paroxysm of vomiting. From the first severe accession, the respiration is hurried and oppressed. As the disease increases in violence, the colour of the whole surface changes to a livid hue; a cold sweat bathes the patient; the hands and feet, and afterwards the whole body, rapidly grow cold. The *facies Hippocratica* succeeds with surprising rapidity. From the early stage when the spasms and other severe affections first set in, many other symptoms are noticed.

"From this period an extreme thirst invariably attends, and, notwithstanding the coldness of the body, there is an ardent longing for great quantities of cold water; which, however, though gratefully and eagerly received, frequently affords no relief to the morbid sensation. An acute pain is felt in the situation of the stomach, which has been generally and distinctly observed to be increased on pressure, and sometimes on inspiration. A sense of heat is likewise felt in the same situation, and often extending over the whole abdomen, which has been compared to the imagined effect of burning embers, or a blister in the stomach. Great oppression and sense of anxiety are also referred to the præcordia. The urine sometimes appears in the early stage, and then it is pale and watery; but, under the existence of the severer symptoms, that secretion, as well as that of bile, is completely suppressed. The tongue is natural at first, but in the course of the disease it becomes furred, and deficient of moisture, and dryness of the mouth and throat is very generally complained of. The hands are sodden with cold sweats, shrivelled and wrinkled, like those of a washerwoman after a day's labour, and frequently of a very dark blue colour. A strong and disagreeable odour, perfectly *sui generis*, is exhaled from the body, and forms another of the striking features of the disease, which once seen must be impressed on the memory for ever." (P. 4.)

The patient often complains of heat, though the temperature of the body is generally diminished. His restlessness and anxiety are also extreme, and this state appears to be attended with an insupportable degree of suffering, and is therefore seldom of long duration: it is gradually relieved, or removed by stupor; frequently a constant inclination to doze is felt, while the patient wakes every minute, tossing and groaning in a dreadful state of anxiety. After an uncertain, but probably *only few hours* continuance of this severity of the symptoms, a remarkable and deceptive change takes place.

"The spasms, the vomiting, and the purging cease, usually about the same time. Whatever is taken into the stomach is retained, even though in large quantity, and clysters not rejected as formerly. About this time, likewise, the above-mentioned state of dozing and stupor appears, and considerably resembles natural sleep: but any favorable inferences which may be drawn from these appearances are quickly contradicted by the continued extreme weakness of the circulation, the coldness and livid colour of the surface, and the deathly expression of the countenance." (P. 5.)

The powers of life continue rapidly to fail; the well-known symptoms of approaching dissolution come on, and death closes the scene, usually (Mr. Orton believes,) with little suffering, for the external and internal senses are completely abolished. When either nature or art is capable of leading the patient through this severe form of the disease, a favorable crisis is generally observable, "which is almost invariably marked by *sleep* of unusual soundness, warm perspiration, and light and natural respiration." This favorable change most frequently occurs before the above-described morbid quiescence is noticed; for this state is very generally followed by death. The patient awakes relieved, and an evacuation of bilious fæces and urine usually takes place. The pulse rises, and other signs of increased action mark the great change which the disease has undergone. The Bengal Medical Board have observed this stage of excitement, or reaction, sometimes rising to a great height, and assuming all the characters of the idiopathic bilious fevers of the country, and occasionally becoming fatal.

Among the natives, a still more rapid and violent description of the disease has frequently been observed. The patient, apparently in health, is attacked with vertigo, ringing in the ears, deafness, and dimness of sight; vomiting and purging of white stools come on, and extreme debility, and even death, are produced in the course of half an hour. Sometimes the disease has been almost instantly fatal. "Several instances were heard of at Hoobly and other places, of natives being attacked with the disease whilst walking in the open air; and, having fallen down, retched a little, and complained of vertigo, blindness, and deafness, they expired in a few moments." Mr. Coates states in the Bombay Reports, that the number of deaths at Punderpoor in a few days were estimated at three thousand, and the patients are described as having been knocked down dead, as if by lightning.

Having adduced this formidable evidence, Mr. Orton, for the first time, expresses the opinion, which in subsequent parts of his work he so industriously and ably supports, that "it is only in the *nervous system* we can reasonably look for an explanation of these occurrences." From the descriptions which have been given by various writers of the more protracted cases of cholera, we learn that inflammation of various organs, especially among European patients, is not an uncommon occurrence; but we are not aware that any other authority than Mr. Orton has been led to the belief that, "in every *lingering* case terminating fatally, there was sufficient reason to believe that inflammation was the immediate cause of death." It is not only,* he says, during the existence of the worst symptoms of cholera that this extreme tendency to inflammation appears, but it may be perceived in a less degree after the favorable crisis and during convalescence. In some enteritis, in others phrenitis, supervened.

"The inflammatory stage will be marked by a want of that increase of the fulness of the pulse which characterizes the other; for, however paradoxical it may appear, it is found that the tendency to inflammation in cholera is nearly in an inverse proportion to the force of the circulation, or at least to the fulness of the pulse." (P. 14.)

It follows, then, that the patient is not safe, although he may have been carried through the chief severity of the attack of cholera, for the remaining general tendency to inflammation is still to be obviated.

Having given a description of the more common and fundamental type of the epidemic, Mr. Orton proceeds to define its different stages, and he dwells earnestly upon the necessity of our attending to the various slight symptoms which occur at the commencement of the disease; for by the prompt application of remedies we may almost with certainty crush it in embryo. It rarely happens that the symptoms of any disease are so constant and unvarying as to enable a writer to give such a description as shall apply exactly to every example of it, and such appears to be especially the case with cholera, although Dr. Kennedy may still correctly remark, that "there is no disease more true to its *general* characters than acute cholera."

Mr. Orton says that the more rapid and violent cases exhibit considerable uniformity, and the latter stages of all fatal cases are

* M. Brierre de Boismont found inflammation of the gastric mucous membrane very frequently in the protracted cases. It is stated also in the Bombay Reports, that, in similar cases, the stomach and intestines generally exhibited appearances of inflammation.— Editor.

very much alike; but, in its less fatal forms, and its earlier stages, the disease presents an infinite variety of appearances.

"It was a common remark among the medical officers at Bellary, that no two cases were found alike. In a considerable proportion there is no appearance of spasm in any part of the system; in many there is no purging; in some no vomiting; and in others, neither of these symptoms. In one class of cases a very considerable morbid exertion of power appears in the muscles both of voluntary and involuntary motion, in the organs of secretion, and in the circulating system: in another class, there is little or no increased action, but on the contrary a general cessation, more or less suddenly occurring, of all the moving powers of life." (P. 16.)

The *milder form* of cholera is next described, and the symptoms in which this form differs from the severer kind are pointed out.

| "CHOLERA MITIOR. | "CHOLERA GRAVIOR. |
|---|---|
| *General character.* | *General character.* |
| Increased action. | Diminished action. |
| *Particular characters.* | *Particular characters.* |
| Excessive secretion of bile throughout. | Entire suppression of bile until the favorable crisis. |
| Violent spasms of the voluntary musles. | Slight spasms, or none. |
| Moderate debility of the animal functions. | Extreme debility of the animal functions. |
| Full and strong pulse. | Extremely weak pulse. |
| Hot skin and flushed face. | Cold skin and sunken face. |
| Violent and frequent retching, spasms in the intestines, and purging. | No spasms in the intestines; not more than one or two evacuations by vomit or stool." |

It is to be observed, that, on the occurrence of a favorable crisis, these distinctions will in a great measure cease. In both there will then be excessive secretion of bile, heat of skin, and a pulse above the natural standard, both in frequency and force.

Mr. Orton details at some length the more infrequent and anomalous symptoms of cholera, but for the information he gives upon this point we must refer to the work. From the appearances he himself observed on dissection in a great number of instances, and from the statements of various writers to whom he refers, we find that a high degree of venous congestion prevailed in the internal organs in general, and that there was a great tendency to inflammation in various parts." The dark colour of the surface of the bodies and of the blood clearly shew a great deficiency of the change which that fluid undergoes in the lungs, and this inference is supported by the appearance of the blood drawn during life, which has constantly been observed to be of a very dark colour." The appearances of the biliary and urinary organs after death confirm the fact evinced by the symptoms during life, that not only the evacuation, but the secretion of both bile and urine are completely suppressed in the worst forms of the disease. Mr. Orton states that "the gall ducts were found pervious:" we learn, however, from other writers, and as one example we refer to Mr. Scot,* that cases of constriction and impermeability of these ducts were equally numerous with those of an opposite character. During a discussion at the Westminister Medical Society, we remember also that Mr. Boyle mentioned the not unfrequent appearance of constriction of the gall ducts. The urinary bladder was almost invariably found contracted to the size of a hen's egg, without containing a drop of urine: this fact is confirmed by the testimony of all writers upon the disease.

Mr. Orton next enters into the consideration of the proximate cause of the disease, and, as far as we can judge from the arguments he adduces, and from the positive statements of, and the inferences to be drawn from the description given of the severe form of cholera by the best authorities, he is justified in concluding, "1, that the proximate cause of cholera consists in a diminution of the energy of the nervous system; 2, that the deprivation of nervous influence thus produced, extends in various degrees to all the functions; and immediately produces the phenomena of the disease."

A mere verbal dispute about the propriety of the term proximate cause is wisely avoided: it is here meant to express the essence of the disease, and the immediate origin of its symptoms.

We cannot follow Mr. Orton through the elaborate and ingenious analysis he introduces of the various symptoms of cholera, for the purpose of shewing the validity of the inferences just quoted; but we must, in justice to him, remark, that this part of his work is as interesting as it is conclusive, and we feel confident that those who were previously inclined to take a different view of the subject will confess themselves, after the perusal of the "analysis" referred to, converted from their former opinions, and convinced of the accuracy of those here maintained.

In the fifth chapter of the work an attempt is made to trace a relation between cholera and the "sweating sickness" and some other epidemics, and certain diseases of the class neuroses. The analogy which exists between the effects of various poisons and cholera can scarcely escape observation; the symptoms from these two causes, indeed, are not unfrequently so identically the same as hardly to admit of distinction, although that distinction is, in many points of view, of the highest importance to the practitioner. Mr. Orton dedicates a few pages to this subject.

We now approach some chapters of the work that may properly be passed over with a very brief notice, and we cannot but regret that the author did not himself greatly curtail the long and even not very interesting discussions therein contained, especially as the subsequent modifications of some of his opinions, as stated in the supplement, render the subjects "of the meteorological occurrences

* Report to the Madras Medical Board.

attending the epidemic," and the "sol-lunar influence" upon the disease of at best but secondary importance.

The tenth chapter contains a "brief sketch of the treatment of cholera." The first object insisted upon is the *early* employment of remedies. SYDENHAM long ago maintained that opium is the sheet-anchor in the cure of cholera, and "such it has been in the treatment of this epidemic, and it has proved itself worthy of the character." Mr. Orton thinks it probable that a single dose of it alone, given at the very commencement of the disease, would be found, in a great majority of instances, to put an effectual check to its progress. The abuse of this remedy, however, is represented as highly pernicious; for, in its secondary effects, and perhaps in the immediate effects of very large doses, it produces all that oppression or abolition of the powers of life which so strongly mark the greater degrees of the disease." If, then, we proportion the quantity of the remedy, or its repetitions, to the danger, we shall probably produce or increase the very affections which we attempt to obviate or remove." Solid opium is recommended in preference to the tincture, as it is less liable to be rejected. To an adult four grains may at first be given.* If favorable symptoms do not appear, the remedy is to be continued in less quantities at intervals of three, four, or six hours, in order to keep up its primary or favorable effects, and to prevent that sinking which appears to follow its use. In the last stages, its effects appear almost always injurious: Mr. O. states that it is not then indicated by any one symptom, except the anxiety and want of natural sleep; and experience has shewn that no quantity of the remedy is sufficient to remove these symptoms under those circumstances. Our chief reliance rests upon the first dose of opium, and if the disease have made some progress, or where the symptoms are very alarming, a quantity somewhat larger than that above mentioned may be ventured upon. Injection, or the introduction of solid opium into the rectum, are also said to be valuable methods of employing this remedy. Dr. JAMES JOHNSON first suggested a combination of opium with scruple doses of calomel, and the best effects have been stated by many practitioners to result from this mode of treatment.

"It must however be observed, that the benefits of this practice have been by others strongly called in question. A practitioner, for whose opinion I have the highest deference, and whose practice in the disease appears to have been more successful than any other which has come to my notice, has never given the calomel until after the favorable crisis." (P. 305.)

The good effects arising from the judicious and moderate employment of stimulants, as brandy, aromatic tinctures, camphor, and essential oils, cannot be doubted: they should be combined

* "Indian opium, which is here alluded to, is considerably weaker than that of Turkey: three grains of the latter may be equal to four of the former."—ORTON.

with the first dose of opium, and their subsequent use must be modified by the state of the patient. Mr. Orton was in the habit of employing a strong tincture of cloves in brandy. A preponderance of inflammatory affections, heat of skin, &c. will point out the impropriety of giving stimuli.

Upon the subject of bleeding in cholera, the greatest contrariety of opinion appears to prevail among those who have had ample opportunities of ascertaining the effect of this and other remedies from their personal experience. One writer tells us to bleed to *produce* reaction, to lighten the load which oppresses the powers of the patient; another assures us that we cannot safely deplete until reaction is positively established. We are inclined to place much confidence in the statements made by Dr. KENNEDY* upon this important practical point, as we find them confirmed by many of the most experienced writers. Mr. Orton speaks more cautiously and less definitively upon the subject.

"Bleeding has been extensively practised in this disease on all the three establishments, and has received the highest encomiums. There is, however, reason to believe that the opinions of medical men are still divided as to its general propriety. My own experience and inquiries have been such as at once to impress me with a high opinion of its good effects, and to shew the necessity of caution and discrimination in its employment; for though its effects have been in general favorable, in some instances they have appeared the contrary. It is scarcely necessary to point out the habits in which this remedy is most applicable; the young, the robust, and plethoric, and particularly the European. It is a measure which is not necessary, and may do harm, in the slighter cases; and though its benefits are doubtless greatest when employed early, yet the lesser evil appears to be to defer it until symptoms arise indicating considerable danger, when it is not called for by symptoms clearly shewing its necessity. In those cases which are marked by highly increased action, it is our most valuable means of preventing the occurrence of the opposite and more dangerous state, and of those highly dangerous visceral inflammations, which in many instances would certainly follow in the European habit, without its use, and often of removing those latter affections when set in. In the latter stages, and in most lingering cases, this remedy, as well as almost every other, is found perfectly inadequate to the removal of the active inflammation which so frequently then prevails. From some accounts of the effects of bleeding in this disease, we might be led to expect the pulse to rise under the operation; but this, I believe, will rarely occur. In my experience, the first effects of the evacuation have been, in this as well as other diseases, a diminution of the force of the circulation; but the vessels, relieved of a load which the disease had rendered them unequal to, quickly regain their tone, and act more vigorously than before.

* Vide our last number, p. 506, 510.

In some instances, however, the pulse has sunk immediately after the bleeding, and never risen again; but in all probability this would have happened in a short time without the operation. Much still remains for future observation to determine regarding the limitations which are necessary in the use of this remedy.* The operation should invariably be performed in the recumbent posture, and care taken to prevent the patient rising after it; as it is obvious those precautions must lessen the danger of producing that sinking which so often follows the remedy. In fact, it is advisable to preserve that posture throughout the disease, for the others are often found in an instant to bring on returns of the vomiting and spasms." (P. 306.)

Counter-irritation should be excited on the surface of the body, as speedily as possible; but Mr. Orton considers it doubtful whether a *rapid* effect is not more desirable than an *instantaneous* one.

Various means have been suggested for restoring the quickly-fallen heat of the body of a cholera patient: it is here stated that "the warm bath, in a reclining posture, will probably be still found one of the best means of restoring heat, and exciting and determining to the surface." We again refer to Dr. Kennedy's opinions,† because we are convinced that the distinction he lays down between the effects of *moist* and *dry* heat are important and correct. To allay the tormenting and excessive thirst, Mr. Orton recommends an infusion of ginger, made palateable with sugar and a little milk.

After the favorable crisis, *pargatives* are chiefly to be depended upon; care being taken to select those which best agree with the stomach. Calomel, from its power of producing copious discharges of vitiated bile and fæces, will be highly serviceable; but "it appears advisable, in general, to avoid its full effect on the mouth."‡

We are cautioned against endeavouring to remove that great

* "In justice to Dr. Johnson, it is necessary to mention that his valuable work on Tropical Diseases (which is in the hands of almost every practitioner in India,) appears to have led to the adoption of bleeding in this epidemic: and the merit which accrues to him from this suggestion is of no common kind; for, according to the unfortunate notions which have so long prevailed, it is probable that few persons would have ventured on a practice of this kind, in a disease whose principal characteristic is extreme debility, without some previous evidence of its propriety."—Orton.

† Vide our last number, p. 511.

‡ The following is an extract of a letter from Dr. Lange, chief physician at Cronstadt, to Drs. Russel and Barry. "At the commencement of the epidemic, a heated iron (*ferrum candens*,) was employed upon fourteen patients, by applying the instrument to each side of the spine of the back, in the region of the lumbar vertebræ. What is very remarkable is, that, at the moment of the application of the heated iron, the spasms immediately ceased, the pulse began to be more perceptible, the heat of the extremities returned, and the disagreeable sensation under the scrobiculus cordis diminished, as well as the vomiting and diarrhœa: among others, there were cases in which the spasms were renewed; in this state the sick themselves begged for the application of the heated iron. Out of the fourteen treated with the hot iron, twelve were cured, and two died in the same day." The beneficial effects of the application of the heated iron to the spine appear to illustrate and confirm Mr. Orton's opinion that the proximate cause of the disease consists in a great depression of the nervous system.—Ed.

debility which follows a severe attack of cholera, by stimulants and "nourishing" diet, as inflammatory attacks are to be feared. "The food should be simple, chiefly vegetable, and in small quantities at a time; and if any stimulating liquors are ever allowed, their quantity should be very small."

When the first edition of Mr. Orton's work was published, he was a non-contagionist, but he now candidly confesses that the march of time and events, the great accumulation of facts, and gradual removal of prejudices, have wrought in his mind the same revolution that they have in so many others. "The opinion of the contagious nature of the disease has been gradually gaining ground even in India, and seems to be the general one of Europe "*Veritas magna est, et prævalebit.*" In support of the belief to which he now so creditably confesses himself to be a convert, Mr. O. adduces many facts noticed by himself and other observers. Into these statements we do not deem it necessary to enter, as in our last number we gave quite sufficient evidence of the contagious nature of the disease.

Mr. Scot was among the first who observed that troops on their march from station to station, or in the field, and persons travelling in India, are particularly subject to cholera. Mr. Orton gives a few pages of his supplement to the consideration of this remarkable and by no means unimportant fact, for which he thus endeavours to account.

"The principal circumstances of difference between troops on the march and in quarters, which appear capable of producing disease, may be stated as follows:

"1. An increase of temperature during the day, usually of 10° or 15°, arising from the change from houses into tents; and,

"2. The same imperfect defence from the cold and moisture of the atmosphere.

"3. A degree of labour, which to soldiers is unwonted.

"4. Exposure to contagion on the route." (P. 397.)

Additional proofs are also brought forward in the supplement of the influence of localities on the prevalence of the epidemic, and Mr. Orton comes to the following conclusions.

"The disease shews an evident preference for the low and level parts of a country, and avoidance of more elevated tracts.

"It is found to prevail most severely in all the moist, close, and filthy parts of any city or neighbourhood.

"It commonly follows the course of rivers, both navigable and unnavigable.

"It prevails most severely in all those countries and places, where, from the prevalence of the endemic fever, malaria is known to exist; and in such situations its epidemic duration has commonly been greatly protracted." (P. 401.)

Many interesting facts are mentioned, which show that the extensive prevalence of various other endemic or epidemic diseases, particularly the malaria fevers, has often accompanied, or immediately preceded or followed, that of the epidemic cholera. It appears, also, that a particular prevalence of the endemic or sporadic cholera generally preceded the great stream of the epidemic, and that the atmospheric cause was capable of producing the disease in its ordinary *non-contagious* form, to some, but a trifling extent, in countries distant from that in which the epidemic originated, independent of imported contagion.

Under the head "of the susceptibility or predisposition of the subject to the disease, and of its exciting causes," much valuable matter is given. The propositions maintained in this section are,

"1st. In every body of people, there is a large proportion incapable of receiving the disease, though exposed to all its usual causes.

"2d. Having undergone an attack of the disease confers a great degree of immunity, at least for a time, from its future attacks.

"3d. The indigent part of society, the old, the weakly, or unhealthy, and the intemperate, are particularly liable to the disease.

"4th. The usual exciting causes of the disease are, exposure to great heat, cold, or moisture, errors of diet, over-exertion, the depressing passions, and in general whatever tends to debilitate or disorder the frame." (P. 433.)

There exists but little difference of opinion as to the identity of the epidemic cholera of India with that of Europe: all the Russian official reports unite in shewing that the disease is one and the same,* and Mr. Orton is also impressed with this belief. He states, however, that the disease has undergone some modification during its long course from the mouths of the Ganges and Megna to Vienna and Berlin; and after having considered the evidence afforded by Drs. Kier, Barry, Russel, and Mr. Searle, he concludes that it is chiefly in the much greater frequency of the occurrence of the *inflammatory* stage, and not in any essential alteration of symptoms, that the epidemic cholera of Europe differs from that of India.

Our analysis of Mr. Orton's work is now completed, and we trust we have given a sufficient sketch of its very valuable contents to enable oar readers fully to comprehend the most important statements it contains. It is much to be regretted that severe illness prevented Mr. Orton from entirely remodelling the contents of the volume, as the reader of it may, from its faulty arrangement, sometimes form an erroneous judgment of his present opinions. In the first part of the work, for example, which Mr. Orton tells us is scarcely at all altered from the state in which it first appeared, the contagious nature of cholera is denied; while in the Supplement we are glad to find that the contrary opinion is maintained. This part of the work also contains some other, but comparatively trifling, modifications of opinions supported in the first edition. In order, then, that the author may not be arraigned for still holding doctrines which he has abjured, it will be necessary carefully to compare the supplement with the first part of the volume.

* Dr. Bisset Hawkins on Cholera, p. 120.

*On Polypi of the Heart as an Idiopathic Affection, and as a Cause of Death.* By William Harty, m.d., Physician to the King's Hospital, and to the Prisons of Dublin. (*Dublin Medical Transactions*, vol. i. part i.)

The subject of this paper may probably be regarded by some physicians as one of more pathological curiosity than practical importance. It has been said that we have no means of relief for the disease, even if we should succeed in our attempts to establish a diagnosis which will clearly indicate the existence of "polypi" of the heart. We fear that this assertion must at present be admitted; but still the investigation cannot be altogether useless if any facts can be elicited, for facts are always worth obtaining, even if they are of no immediate practical utility. But it would be of immediate advantage to the physician to be enabled to ascertain the existence of polypi of the heart as an idiopathic affection; for this addition to his knowledge would occasionally be a sure guide to his prognosis. To know that a patient is labouring under an irremediable disease, must be mortifying and distressing to the practitioner, but still his acquaintance with the fact is of obvious and great importance.

It is not, perhaps, worth while to enter into a dispute respecting a term that has been long applied, and generally received, to designate those fibrinous concretions which are occasionally found in the heart and large vessels; but we may remark, that the word "polypus" is at least fantastical, and derived from a forced and imaginary resemblance supposed to exist between such concretions and certain marine zoophytes. We shall continue, however, to employ the term as it is commonly understood.

Some pathologists have doubted whether polypi of the heart ever exist as an original and essential disease: they have been said to be always dependent upon some disease of the heart itself,* or to be formed by coagulation of the blood during the last moments of expiring life, or immediately after death. We are inclined to believe that these concretions do *occasionally* exist as idiopathic affections, although we willingly admit that polypi of the heart, as they are termed, are generally accompanied by some structural disease of the organ, and that, in most instances, they are formed either just before or immediately after life is extinct. We cannot yet venture to declare that any certain diagnostic signs have been detected which will enable the physician to ascertain the existence of these polypi during life; but it is certainly not difficult to form a tolerably positive opinion, from their examination after death, whether they have long existed, or whether they are of very recent origin. Two kinds of these polypous concretions are found in the heart or large vessels, each possessing very different characters: those which it may be presumed are formed after death, or during

* Dict. des Sc. Med., tom. 44, art. "Polypiforme."

the last moments of life, appear externally like loose jelly;* they are of a yellowish colour, transparent, and either do not adhere at all, or very slightly, to the surrounding parts; it is only in their centre that there is any appearance of fibrine. They appear to be the result of coagulation of the blood in the cavities of the heart or large vessels. The appearance of the other species is very different, and must, we think, lead to the conviction that they have been formed during life, and have existed for a considerable period: they are of a firm structure, opaque, and of a white colour; adhere so strongly to the adjoining parts that they can only be removed by dissection, and have been found highly vascular and organised.† We can only at present refer to one example in which these polypi have been injected, but there are other similar facts on record.

M. Rigacci, of Florence, has recorded a case‡ in which a well-organised polypus was found after death in the heart. The patient had been supposed to be labouring under an aneurismal dilatation of the left ventricle. Upon dissection, a body of a fleshy appearance, similar to sarcoma, was found in the left ventricle. On the external surface of the polypus were seen three reddish fillets, which, arising from the carneæ columnæ, extended to the morbid production, and appeared to be lost in its substance: these, examined with a good lens, were found full of reddish fluid, and were recognised as sanguiferous vessels. To prove this important and unanswerable fact the more satisfactorily, two of the fillets were injected with mercury: one of them burst, but the other was completely filled, and exhibited its divisions and ramuscules, which were lost in the substance of the polypus.

Allan Burns relates a case in which a polypus adhered very firmly to the internal surface of the left ventricle, and a small abscess, containing well-formed purulent matter, was found in its substance. In another instance, Burns states that the polypus was firmly attached to the interior surface of the left auricle, and there were slight osseous depositions found in it, and some of its vessels could be injected. In a third case recorded by the same writer, the polypus in the heart had a strong resemblance to nasal polypus, and was strongly attached to the internal surface of the right auricle. In all these cases the heart was organically diseased; but, as Dr. Reeder§ observes, "the deposition of osseous matter, the inflation of some vessels, and their having strong attachments, seem to favor the idea of these having been living polypi."

Additional facts might easily be collected from various other authorities, which prove that "polypi" of the heart do not *always* consist merely of unorganised coagulable lymph, and that they are

* Dict. des Sc. Med., tom. 44, art. "Polypiforme."

† Edinburgh Med. and Surg. Journal, April 1817, p. 182; and Andral's Pathological Anatomy, translated by Townsend and West, vol. ii. p. 350.

‡ London Med. and Phys. Journal, September 1829, p. 268.

§ On Diseases of the Heart, p. 212.

not *always* formed in articulo mortis, or after the extinction of life: but we must now proceed to the evidence contained in the paper before us.

Dr. HARTY first refers to the opinions of CORVISART, who admits that polypi of the heart may be, and frequently are, the cause of death. In proof of this assertion, he gives the details of two dissections in which such concretions were formed, firmly adhering to the fleshy pillars of the right ventricle in one case, and of the left in another; the pillars themselves being entirely destroyed where it had adhered in the latter case, proving that it had been "formed there long before death." Corvisart adds, that on many other occasions he had seen these concretions, usually of a yellowish white, and of a fibrinous structure, so consistent, tenacious, and intimately adhering to the internal fibres of the heart, that he could not but admit as a fact, proved by experience, the formation of these concretions long before death, and the presence of which he had sometimes announced before opening the body, from the symptoms of the previous disease. It is strange that Corvisart has not enumerated these symptoms.

Dr. Harty relates two cases, which it was "his misfortune to encounter before the stethoscopic era had commenced in Ireland: these cases appear to him well calculated to establish two positions;" first, the undoubted existence of such concretions, not only *as a cause of death*, but as the source of independent and idiopathic disease; and, secondly, the determination of a diagnostic sign or signs, by which the occurrence of the disease may be surmised with some certainty, even without the highly valuable co-operation of the stethoscope." The following is a condensed account of the cases related by Dr. H., but we have been careful to omit no part of his statement which touches upon the points he designs to illustrate.

CASE I. Miss R., aged fourteen, of slight frame and narrow chest, had for several years been subject to repeated attacks of chorea, all of which had yielded to active and long-continued purging. In December 1814, symptoms of chorea returned. While in this state, and before Dr. H. had been consulted, a very violent storm occurred in Dublin, by which Miss R. was roused from her sleep: she rushed from her bed in extreme agitation, thought the house was falling, and a long time elapsed before she regained any degree of composure. The symptoms of chorea were aggravated, and a state of fever, with palpitation and hurried respiration, followed this excessive alarm. These latter symptoms abated, and Dr. H. was not again consulted till the middle of January, by which time the chorea had made further progress, although the purgative plan had been again pursued. The urgent symptoms in January were, besides those peculiar to chorea, a febrile state, with a pulse at 112; pain of the chest and left side; delirium at night. Palpitation existed, but was little complained of. These symptoms were relieved by bleeding to twelve ounces, and by free purging, the fæces being first black, then mud coloured. Both the chorea and the febrile state had much abated, until imprudent exposure to the weather in February re-excited the fever, and aggravated the chorea. These symptoms again yielded to a second venesection, and a repetition of the other remedies.

The patient continued in a favorable state until she was attacked with sore throat, about the middle of March, when the symptoms of chorea had nearly ceased: there was then much febrile heat, delirium at night, vessels of the head much distended; some dyspnœa, with palpitation; pulse 120, and full; pain of epigastric and right hypochondriac region, on pressure; alvine discharges highly bilious. She was a third time bled to twelve ounces, and for the first time the blood was buffed. From James's powder, calomel, and other purgatives, speedy relief was experienced: pulse fell to 100; skin was moist; sleep good; no dyspnœa, and the action of the heart, though sometimes violent, was never troublesome. Digitalis, with purgatives, was given, and strict regimen enforced.

Dr. H. did not see the patient again till the 2d of April. She had been again exposed to cold, and was attacked, in the night of the 31st March, by fever, dyspnœa, violent palpitation, œdema of the face, fulness of the abdomen, the pulse (as her mother stated) being intermittent and irregular. When Dr. H. saw her, the pulse was 128, and regular; there was dyspnœa, without cough; constant palpitation; œdema of the face, with a papulous eruption; skin hot; urine scanty, and high coloured.

3d. Breathing somewhat relieved by bleeding and a blister; pulse 120; skin very hot during the night, followed by profuse perspiration; fæces dark.

4th. Has had a good night, with profuse perspiration; breathing quick, but not oppressed; œdema less, and confined to the left side of the face, *on which she chiefly lay;* pulse 116, regular, and full; fæces improved in appearance; urine more copious; and now, for the first time, a short cough, "affecting the throat," began to teaze her.

5th. Passed a bad night from incessant cough and oppressed breathing; palpitation violently increased by lying on left side, and agitation by the application of leeches. At noon, the breathing was quick, but less oppressed; alæ nasi distended; pulse 118, full, strong, and regular. At night, it fell to 108; the leechbites, under the violent influence of the palpitation, bled most freely during the whole day; cough and breathing much easier; slept quietly for an hour, but, on awaking, the breathing oppressed and face swelled; palpitation much relieved; free from pain. In the evening of the 6th, pulse 120, hard, "*vibrating, communicating a peculiar thrilling, whizzing sensation to the finger, on touching any, the minutest, artery in the body;*" respiration very quick; skin hot; face swollen. V.S. ad lb. i.; blood buffy; after V.S. pulse 112, and softer.

7th. Pulse 120. A pint of blood was taken away in the morning, and again in the evening. The pulse was still unaltered; strength and voice good; coagulum of blood firm and buffed.

8th. Symptoms milder: respiration less quick during sleep; motion of alæ nasi much diminished; pulse 106, and hard; skin hot and dry. V.S. ad ℥viij., which was well borne, and the pulse became softer.

9th. Has had a bad night from vomiting, restlessness, and cough; was thought to be dying; respiration was panting, but now much easier, thirty-eight in a minute; pulse 108, and of tolerable strength; *lies perfectly horizontal;* cough strong; palpitation greatly increased by *erect posture.*

10th. Restless and uneasy during the night, though drowsy, and occasionally delirious; can lie on both sides, but still in the horizontal position; breathing more laborious; cough strong; strength considerable; pulse 120, still strong, *and uniformly imparting to the finger the sensation above described;* eyes dim; facies Hippocratica. About five o'clock the patient sat up, and called with great strength to make water, and, on being laid down, instantly expired.

Permission was obtained, about twenty hours after death, to make a *single* incision in the thorax.

"The lungs were sound; no adhesion whatever, and very little effusion in the cavity. Lungs and heart much limited in space by the liver, which was of considerable size, extending upwards into the thorax, downwards to the umbilicus, and also far to the left side. The pericardium exhibited some little appearance of inflammation; it contained about six ounces of clear serum, without any coagulable lymph. The heart itself was enlarged, with increase of muscular substance, the vessels on its surface much distended; a small incision effusing a good deal of blood. Having (though strictly watched,) succeeded in removing the heart itself, with a portion of the large vessels, the following appearances presented themselves to view on opening its cavities: a distinct polypus of a whitish colour, unconnected with any coagulum, nearly filled the right ventricle and auricle, its branches extending into the great vessels, one branch being more than eight inches in length; the whole polypus adhered so slightly, as to be readily drawn out by the fingers; but a thick membraneous substance of the same colour adhered with much firmness to the external side of the ventricle, penetrating into its interstices, and, by means of both membrane and polypus, the valves were bound down, and must have been altogether impeded in their action; both auricle and ventricle were of a vivid colour, and of an inflammatory aspect. The left ventricle and aorta, however, presented a far more singular phenomenon. The ventricle was divided into two nearly equal cavities by an adventitious whitish membrane, firmly adhering to the internal apex, and to the sides of the ventricle in a line nearly parallel to the septum, and terminating, as it approached the aorta, in a rounded organised polypus, tapering to a point, and entering above an inch into the aorta, which communicated very obliquely with the ventricle, the two cavities into which the ventricle was thus divided communicated with each other very partially, where the membrane terminated in the rounded polypus concretion. The side of the membrane towards the left auricle was uneven; towards the aorta, smooth. That auricle had the same inflammatory appearance as the right, and its valves were impeded by membraneous layers, as those of the aorta were by the polypus: three of the carneæ columnæ were much enlarged, one of them being more than twice the size of a goosequill." (P. 228.)

Having concluded his account of this case, Dr. Harty asks,

"What was the nature and extent of the injury inflicted on the heart, in the first instance, by the terrors of the December storm? Was it then a general or partial affection of the organ, or was the nucleus of the disease then formed? Is there not, under all the circumstances of the case, reason to conclude that the polypus membrane of the left ventricle had priority over that of the right, and that the great aggravation of the symptoms, together with the peculiar sensation imparted by the pulse, was caused by, or was concurrent with, the extension of the polypus into the aorta? Was inflammation or hypertrophy of the heart a cause or an effect of the polypous concretions?" (P. 231.)

(To be concluded in our next.) p. 147.

---

*Elements of Surgery.* By Robert Liston, Fellow of the Royal Colleges of Surgeons in London and Edinburgh, Surgeon to the Royal Infirmary, Lecturer on Surgery, &c. *Part Second.*—8vo. pp. 334. Longman, London.

Mr. Liston is so well known to his brethren as an able and experienced surgeon, that it cannot be necessary we should protect his professional character from the danger which it might incur, if he were less "known to fame," on account of the title of his work. Elementary works are so frequently undertaken by those who have no other qualification for the task than that of having attentively studied what others have written upon the subject of them, that they are seldom presumed to be more than mere compilations from the labours of preceding writers. That such is not the character of the present work will be presumed from the reputation of its author. Mr. Liston, of course, says much that has been said before, and so must every author on the "Elements of Surgery;" unless, indeed, all the fundamental principles of the science which had been taught before could be proved to be erroneous: but each subject that he considers is enriched with observations which have resulted from his personal experience, and illustrative cases which have occurred in his public or private practice, are frequently related. Disputed and doubtful doctrines are discussed by Mr. L. with a clearness and convincing confidence to which the humble compiler can never attain; and when he allows former opinions to remain unaltered, his acquiescence in them is evidently the result of a conviction of their accuracy, emanating from his own experience.

The first chapter contains a good deal of interesting matter on "Injuries of the Head." We shall confine our analysis to those points upon which Mr. Liston differs from most other surgeons, and to the practical illustrations he has drawn from his own observation.

"*Separation of the dura mater*" from the cranium, with more or less extravasation of blood between, sometimes takes place as a consequence of blows on the head, even though not severe. The blood may be absorbed, or an unhealthy abscess may form between the bone and membrane, attended with violent, dangerous, and (if neglected) fatal results. The internal mischief is not without external marks of its occurrence. If the scalp is undivided, a puffy tumor forms; and when it has been injured, the wound degenerates, its surface is pale, the discharge gleety, and the exposed bone white and dry. It is also preceded by general disorder of the system, by restlessness and fever; there is sickness, occasional vomiting, shivering, pain of the forehead and back of the neck; in some cases, delirium and convulsions, and perhaps partial paralysis, and ultimately coma. The following cases are adduced to shew that all these symptoms may exist without indicating precisely either the existence or the site of abscess.

"A middle-aged man was brought intoxicated into the Royal Infirmary, with a lacerated wound of the scalp over the upper part of the occipital bone, on the right side of the mesial line. For thirteen days after the accident he did well, walking about the wards in good health, with the wound healing kindly; but on the fourteenth he became affected with hot skin, restlessness, slight incoherency, severe pain in the head, and intolerance of light, with a full, but not quick pulse. A vein was opened, but after three ounces of blood had flowed, he was seized with rigors, vomiting, and violent convulsions; and these symptoms again occurred after the application of leeches to the head. The pain of head and shivering continued, notwithstanding he had been bled to sixteen ounces on the fifteenth. Rigors returned at various intervals, and stupor supervened and gradually increased. He became delirious on the eighteenth. A considerable part of the bone was exposed and dead, and there was a puffy swelling of the scalp around the wound. On the nineteenth he lay insensible. A portion of the dead bone was removed by the trephine, and the dura mater was found covered with lymph, but no appearance of effused blood or pus could be perceived. He suffered little from the operation, but continued insensible, passing his urine and fæces in bed, with dilated pupils, quick breathing, and subsultus tendinum: his pulse, which had previously never been above eighty, now became one hundred. He died on the morning after the operation.

On dissection, the right hemisphere of the brain was found of the healthy appearance; but four ounces of pus lay over the left hemisphere, between the dura mater and arachnoid, which latter membrane was of a granular appearance; there was also a small sloughy spot of the dura mater over the left anterior lobe.

"A woman, aged forty, fell down and sustained a wound of the scalp on the upper part of the occipital bone on the left side; she suffered but little from the accident, and continued to live freely and irregularly. Seven days after the injury she was seized with shivering, and on the ninth day she lay comatose, voiding her fæces and urine involuntarily. The wound was pale and gleety, and the surrounding scalp puffy; the bone was bare and white; pupils dilated; pulse eighty. The trephine was applied, and fluctuation felt beneath the exposed dura mater, which was otherwise unchanged in appearance; the membrane was divided by a crucial incision, but only a small quantity of bloody serum escaped. Shortly after the operation she became quite sensible, but again sunk into a state of stupor, with slightly stertorous breathing and contracted pupils. However, all traces of coma disappeared next day, and she recovered soon and perfectly, apparently without having received either benefit or injury from the operation of trephine." (P. 19.)

*Fractures of the base of the skull* are the result of great force applied to the lateral parts of the head, to the vertex, or to the base itself, through the spinal column. A blow inflicted by an obtuse body on the top of the head whilst it is at rest and fixed has the effect, by producing expansion of the lateral parietes, and forcing the base down upon the upper part of the spinal column, of breaking up the connexions of the bones at the base, which is the weakest part of the cranium, and splintering them to a greater or less extent.

"Again, if a person falls from a height, he perhaps alights on some part of his trunk, as the buttocks, and this coming to a state of rest, whilst the head is still in projectile motion, the spinal column is driven towards the cavity of the cranium, and the same effects are thereby produced as in the preceding instance. Or the patient alights on his head, and the base of the cranium is then impinged upon by the weight of the whole trunk, as well as by the force of the projecting power, and in this case also the base is frequently broken up. Concussion has resulted from falls when the person has alighted on his nates or feet; but the symptoms attendant on fracture of the base are more generally those of compression of the brain. In this accident the bones are seldom displaced to any great extent; the dura mater is generally lacerated, its blood-vessels, and frequently its sinuses, are wounded, and blood is consequently effused at the base of the brain, which is the most important part of that organ. The upper part of the brain may bear pressure to a considerable degree without bad consequences ensuing, but compression at the origins of the nerves is always highly dangerous, and generally fatal. Bleeding from the nose, mouth, and ears has been considered, when attended with other circumstances and symptoms evincing a receipt of violent injury and consequent cerebral disturbance, as decisive of fracture at the base having occurred. But we find that such bleeding happens in slight injuries unattended with any circumstances or consequences to induce a belief that so serious an injury has taken place: and again, in cases where dissection has shewn most extensive fracture of the temporal, sphenoid, and æthmoid bones, no blood had issued from their external openings. Fracture of the base of the skull generally proves fatal, but many cases are met with in which there is reason to believe that it has taken place, and yet the patients recover, perhaps with partial paralysis. Of this I lately met with a good example in the case of a girl seven years of age, whose head had been squeezed between a wall and the back of a cart, and thereby considerably flattened. She lay insensible for several days, with all the symptoms of compression, and with blood flowing in small quantity from the nose, mouth, and right ear. An extensive abscess formed over the right temporal bone. She ultimately recovered, but remained affected with paralysis of the right side of the face and amaurosis of the left eye; sensation in the paralysed parts being quite perfect." (P. 27.)

Most surgical authorities lay it down as a practical axiom that the operation of trephining is rarely, if ever, justifiable, except on account of the existence of urgent symptoms indicating pressure of the brain. Upon this point we might cite many of the most eminent surgical writers, but the opinion of one will be sufficient for our purpose: we select Mr. Lawrence. He says in his lectures,* "that in the present day we have entirely discarded the doctrine of employing the trephine, or instrument of any sort, in all cases of fracture of the skull, and consider it as a measure that we are not to have recourse to, except where there is depression of the bone, and *that depression of the bone accompanied with symptoms of pressure on the brain.*" "In all cases of fracture with depression, whether the depression be little or whether the depression be great, unless symptoms be present indicating pressure on the brain, it is not judged right to proceed to the operation of trephining it." In the Hôtel Dieu, the unfavorable results of trephining were so numerous, that Desault had entirely abandoned the operation for several of the last years of his life. Mr. Lawrence, from what he has seen at St. Bartholomew's Hospital, is "nearly" inclined to coincide with Desault: he says,† "that of the instances in which I have seen the operation performed in this hospital, the greater number have terminated fatally, so that I can cite to you, as far as the experience of this hospital goes, very few instances in which the life of the patient has been saved by the operation of trephining. Mr. Liston, however, maintains a different doctrine; he says,

"Injuries of the cranium inflicted by sharp bodies, such as divide the scalp and cause compound fractures, are generally attended

* Med. Gazette, vol. vi. p. 599. † Ibid.

with splintering of the internal table, and require the trephine. The existence of this sort of fracture of itself, without a single bad symptom, without any present disturbance of the sensorial functions, is a sufficient warrant for the application of the trephine, so as to permit the removal of the detached portions of the inner table: and this should be done before inflammatory symptoms have shewn themselves. The brittleness of the internal layer of the skull is well known. In fractures inflicted with sharp and pointed instruments, as a bayonet or pike, the corner of a sharp stone, or the heel of a horse's shoe, the external opening is often very small, it is a mere puncture; in the bone there is a central depression, from which fissures proceed around in a radiated form, and hence the accident has been termed punctured, or starlike fracture. But though the external wound is apparently insignificant, the vitreous table is extensively separated, and, perhaps, broken into innumerable minute and sharp spiculæ. These sharp portions are driven down upon the dura mater, and by them the membrane is often severely lacerated. If these are not removed soon after the accident, inflammatory action is almost invariably lighted up on the surface of the brain; and we cannot expect to allay or avert such action by general antiphlogistic means, however energetically applied, so long as their exciting cause remains. It is in such cases, I repeat, that the operation of trephine is imperiously called for. Sometimes, however, patients are found to recover from punctured fracture of the cranium, without the operation having been performed, as in the following case, the only one so terminating which I have met with: On the 4th September, I visited a gentleman, aged thirty-five, who had received a punctured fracture of the cranium, on the 29th of August; a heavy fork had fallen from the top of a haystack, and struck him on the upper part of the head. Immediately after the accident, he became confused, but not insensible; he lost the power of motion in the right lower extremity, but almost instantly regained it. Next day the right arm became weak, and when I saw him, he was almost wholly unable to move it: he could not bend his fingers, nor raise the arm, and retained but very slight motion in the elbow joint. There was a small wound of the scalp, nearly healed, over the posterior part of the left parietal bone, close to the sagittal suture, and nearly midway between its two extremities. A probe passed down to, and through the bone; and there was slight swelling of the scalp around the wound. He had felt pain in the right ear, and in the forehead whilst stooping, for some days after the accident. No blood had ever escaped from the ear. A fit of shivering occurred on the night following the injury, but never returned. He soon recovered completely.

I subjoin a case of an opposite description. A coachman was knocked down, late on a Saturday night, and fell with his head on the corner of a stone at which masons had been recently working. After being carried to his lodgings, he recovered from the stupor produced by the combined causes of liquor and blows; and next morning he went to have his head dressed by an apothecary, who extracted a fragment of a stone with difficulty from the wound of the head. The patient then drove a party to church, and probably drank some more whisky during the day. He afterwards felt indisposed, and was seized with sickness and shivering in the afternoon. On Monday he was in a violent fever, and I saw him in the evening. He had been delirious, but was now lying in a state of stupor. There was a hole in the right parietal bone, capable of admitting the point of the little finger, and many loose fragments of bone were felt lying on the dura mater: a trephine was applied, and numerous spiculæ removed. Afterwards the circulation became much excited: he was bled copiously, and antimony was exhibited in nauseating doses, but he died early on Wednesday morning. On dissection, there were found marks of violent inflammatory action on the surface of the hemispheres. The vessels were unusually numerous and highly engorged, and lymph and pus were effused in considerable quantity; the arachnoid was opaque, and the cerebral substance was somewhat softened. Had the operation been performed at an earlier period, there is every probability that the inflammation, which proved fatal, would have been averted, as in the following instance: A quarryman received a blow from a sharp stone of considerable size, which rolled down a precipitous bank, and struck him on the vertex. He lay insensible for half an hour, but recovered, and followed his occupation during the rest of the day. In the evening he came for advice. There was a small wound in the scalp, and the subjacent bone was fractured in the same manner as in the former instance, but he felt no uneasy symptoms whatever. The consequences likely to result from such an injury, and the necessity for trephining, were represented to him: he agreed, and the operation was performed on the spot. Many sharp fragments of the inner table were extracted: he proceeded home, never had a bad symptom afterwards, and consequently required no treatment, save dressing of the wound." (P. 30.)

Mr. Liston believes that, if the operation of trephining be undertaken early, it will, in all probability, succeed in averting future evil, more especially if the dura mater be not wounded. As a proof of the unfavorable nature of this latter circumstance, the following case is given.

"A young man, aged eighteen, received a kick on the forehead from a horse, September 9th: he remained perfectly sensible, and did not fall to the ground. Shortly after, he was seized with vomiting, which recurred at intervals; his pulse was regular, but feeble; pupils dilated. On the centre of the forehead there was an irregular wound, which extended to the root of the nose; and, on introducing the finger, the os frontis was found fractured, and a small portion of it comminuted and depressed. The trephine was applied, and several detached portions were removed, with some difficulty, from beneath the undepressed portion of the bone. A

spicula had lacerated the dura mater, and penetrated the substance of the brain to the depth of half an inch: on removing it, a small portion of cerebral matter escaped. The fracture extended apparently in the direction of the right orbit. In the afternoon the pulse was sixty-four, of good strength, and the pain in the wound had slightly increased; he was bled to fourteen ounces, and ordered the antimonial solution. Afterwards, the pain of the head increased, the pulse rose, the scalp around the wound became the seat of puffy swelling, and several small abscesses formed: the antiphlogistic regimen was rigorously followed, and the abscesses were freely opened as soon as they began to form. On the 21st, a portion of the brain had sloughed, and there was some appearance of fungus cerebri: an incision was made into a swelling over the right temporal muscle, and ℥viij. of blood allowed to flow. On the 22d, several portions of brain were discharged, and the pulse was 100, and intermitting. Next day, he was delirious, and a hernia cerebri protruded, of a sloughy appearance and considerable size; pulse 142. Soon afterwards he became comatose, and died early in the morning of the 23d. On dissection, the integuments and pericranium surrounding the aperture in the frontal bone were found much thickened, and infiltrated with pus and serum. The dura mater at the wound was of a sloughy appearance. There was great effusion of purulent matter under the dura mater investing the right hemisphere of the brain; the corresponding tunica arachnoidea was thickened and opaque, and between it and the pia mater there was considerable deposition of lymph and pus. The fungus was collapsed, of a dark colour, soft consistence, and connected with the anterior lobes: the surrounding cerebral matter was much softened, and mixed with pus. The fracture extended through the orbitar plate of the right os frontis, over which lay two small spiculæ of bone; and a similar fragment was situated over the right optic nerve." (P. 33.)

It will not be expected that we should formally enumerate every subject treated upon by Mr. Liston in this work: the title of it sufficiently indicates its comprehensive nature. We assume, then, the liberty of passing over many chapters upon various surgical diseases. We stop for a moment to observe, that Mr. Liston has omitted to caution the young surgeon against an accident which has more than once occurred in the performance of that very simple operation, *division of the frænum linguæ in infants*. "The operative procedure is simple and safe: the tongue is raised towards the palate, either by a spatula or split card, or, what is better, by the fingers, and the frænum is cut across to a sufficient extent by blunt-pointed scissors." As Mr. L. addresses himself chiefly to students and junior practitioners, it would have been well to remark that this operation is attended with danger when unskilfully performed. The raninal arteries have been occasionally wounded in dividing the frænum linguæ; and Mr. Cooper states "that the records of surgery furnish us with proofs that the mere bleeding from the raninal veins and the small vessels of the frænum may continue so long, in consequence of the infant's incessantly sucking, as to produce death."* Another accident, sometimes following too extensive a division of the frænum, consists in the tongue being thrown backward over the glottis into the pharynx, where it lies fixed and causes suffocation.† The subject may appear, perhaps, too trifling for any comment, but we must observe, that carelessness on the part of young and inexperienced operators is never more to be guarded against than in the performance of operations which seem too insignificant to demand any particular attention.

In speaking of *wounds of the neck*, Mr. Liston remarks, that when, from the untoward circumstances of the case, or from neglect, an opening in the windpipe remains long open, and becomes fistulous, the larynx contracts, and the voice is in a great measure lost; the patient breathes almost entirely by the unnatural opening, and all the respiratory functions are imperfectly conducted: but even this state of parts may admit of remedy, as is exemplified by the following case.

"Elizabeth Oswald, aged twenty-seven, attempted suicide in 1826, and wounded the larynx through the crico-thyroid ligament. She was under treatment for several months; but was at length abandoned with loss of voice, breathing entirely through a silver tube placed in the original wound. On her applying to me, I found the larynx had contracted; an exceedingly minute aperture, not capable of admitting a common probe, extended from the wound towards the glottis, constituting all that remained of the upper part of the natural air-passage. Bougies, the size of darning needles, were introduced from the wound into this diminutive canal; and by gradually increasing their bulk, the passage was brought to its natural diameter in less than three months. Part of the trachea below the wound had also contracted considerably, and was dilated by similar means.

"A long œsophagus tube was introduced by the wound into the mouth, there laid hold of and drawn upwards, and then pushed down into the trachea, so that it extended from the mouth to some inches below the wound of the trachea. Its introduction was followed by a severe fit of coughing, which lasted about half an hour. The tube, nine inches long, and equal in diameter to the largest œsophagus tubes, was retained in the windpipe for fifteen days, during which it caused great salivation; the teeth loosened, and the strength was extremely reduced.

"The callous edges of the wound were removed by incision, and the opening closed by suture. The tube was removed on the tenth day thereafter, and the patient breathed well. Soon, however,

* Surg. Dict. p. 539; 6th edition.

† Petit, Traité des Maladies Chir. t. iii.

respiration became difficult, and tracheotomy (below the isthmus of the thyroid) was performed. A silver tube was introduced, and retained for five days, when it was replaced by a smaller one. After twenty days, the tube was removed altogether, and in a short time afterwards the wound closed completely. The patient continued to breathe with ease through the larynx, and slowly recovered her voice. When agitated, or after sudden and violent exertion, her inspirations are a little longer than natural, but in other respects the cure is complete." (P. 243.)

In the chapter on the *diseases of the windpipe*, the following instance is related of severe dyspnœa caused by external violence.

" A fine healthy child, aged eight, in running across the street, fell, and struck the larynx with great force upon a large stone. She was taken up quite lifeless, and some time elapsed before respiration was at all established. A gentleman, finding her face livid, opened the temporal artery, and applied leeches to the throat, with some relief. I saw her about three hours after the accident. The breathing, inspiration more particularly, was exceedingly difficult; and this appeared to proceed not only from the injury to the larynx, probably occasioning loss of power in the muscles, but from the collection of some fluid in the trachea and its ramifications. The child was evidently in such a state, unless active measures were resorted to, and that speedily, a fatal termination would soon take place. Tracheotomy was performed; a quantity of coagulated blood and bloody mucus was evacuated from the opening; and when the discharge and coughing had ceased, a tube was introduced. In a short time the tube was withdrawn, the aperture closed; and no unfavorable symptoms recurred." (P. 257.)

We may very fairly extend the approbation with which we spoke of the first part of these "Elements of Surgery" to the present continuation. The third and concluding part is advertised as being in active preparation, and, when the work is completed, it will form a very proper preliminary study for surgical students, if we may judge from the merit of the two volumes already published.

